# A Pilot Randomized Control Trial of the Striving Towards EmPowerment and Medication Adherence (STEP-AD) Intervention for Black Women Living with HIV

**DOI:** 10.1007/s10461-024-04408-w

**Published:** 2024-07-16

**Authors:** Sannisha K. Dale, Ian A. Wright, Aarti Madhu, Rachelle Reid, Naysha N. Shahid, Mya Wright, Jasmyn Sanders, Arnetta Phillips, Allan Rodriguez, Steven A. Safren

**Affiliations:** 1 Department of Psychology, University of Miami, 5665 Ponce de Leon Blvd, Miami, FL 33146 USA; 2 Department of Economics, Miami Herbert Business School, University of Miami, Miami, FL USA; 3 Clinical Immunology, Infectious Diseases, University of Miami Miller School of Medicine, Miami, FL USA

**Keywords:** Black women, HIV, Medication adherence, Trauma, Intervention, Cognitive behavioral therapy

## Abstract

Black women living with HIV (BWLWH) face adversities associated with lower HIV medication adherence, viral non-suppression, and mental health symptoms (e.g., post-traumatic stress disorder) such as trauma/violence, racism, HIV-related discrimination/stigma, and gender-related stressors. We developed the first intervention based in cognitive behavioral therapy and culturally congruent coping for BWLWH to increase medication adherence and decrease PTSD symptoms by enhancing resilience, self-care, engagement in care, and coping for trauma, racism, HIV-related discrimination/stigma, and gender-related stressors. A pilot randomized control trial was conducted with BWLWH and histories of trauma who were at risk for their HIV viral load remaining or becoming detectable (i.e., below 80% medication adherence, detectable viral load in the past year, and/or missed HIV-related appointments). 119 BWLWH were assessed at baseline and 70 met inclusion criteria, completed one session of Life-Steps adherence counseling, and were randomized to either nine sessions of STEP-AD (Striving Towards EmPowerment and Medication Adherence) or ETAU (enhanced treatment as usual consisting of biweekly check-ins). Women completed a post intervention follow up assessment (3 months post baseline) and 3-month post intervention follow-up (6 months post baseline). Via STATA the difference-in-difference methodology with mixed models compared STEP-AD to ETAU on changes in outcomes over time. BWLWH in STEP-AD compared to E-TAU had significantly higher ART adherence (estimate = 9.36 p = 0.045) and lower likelihood of being clinically diagnosed with PTSD (OR = .07, estimate = − 2.66, p = 0.03) as well as borderline significance on higher CD4 count (estimate = 161.26, p = 0.05). Our findings suggest preliminary efficacy of STEP-AD in improving ART adherence, mental health, and immune function.

## Introduction

In the U.S., HIV is the eighth leading cause of death for Black women (ages 35–44) [1]. With access to active antiretroviral therapy (ART) and a high level of adherence (80–90%) [2], HIV is a manageable, chronic condition and viral suppression can be achieved. However, in samples of predominately women of color the estimated adherence rate is 45–64% [3] and there is a higher risk of virologic failure among Black women compared to White and Latina women [4]. Due to interlocking systems of oppression Black women living with HIV (BWLWH) face multiple stressors including trauma/abuse, racism, HIV-related discrimination and stigma, and gender related stressors [5–7]. These stressors may cause, exacerbate, or co-exist with mental health struggles such as post-traumatic stress disorder (PTSD), depression, and substance use [8, 9]. Collectively these intersectional stressors and mental health struggles are linked to lower HIV medication adherence and viral suppression [10, 11].

High rates of abuse/trauma and PTSD among women living with HIV (WLWH) have been noted consistently in the literature. For instance, over 67% of WLWH (predominantly Black) in the Women’s Interagency HIV Study (WIHS), reported histories of physical, sexual, or emotional abuse [12, 13]. Similarly, a meta-analysis of WLWH [15], found a 55% rate of intimate partner violence and estimated a 30% rate of recent PTSD. These histories of trauma/abuse are related to medication nonadherence, antiretroviral failure, and increased mortality [13–16].

In addition to trauma/abuse, BWLWH also experience racism- and HIV-related discrimination. For instance, among a sample of BWLWH [17] 85% of women reported at least some discrimination “a few times a year” (92% of whom attributed to race, and 87% to gender). Researchers [18] found that African-American women who reported HIV discrimination reported more stress and depressive symptoms, lower self-esteem, and were less likely to seek medical care for HIV. Racial and HIV-related discrimination have also been associated with higher PTSD symptoms [19], barriers to HIV care [20], and depressive symptoms and diagnosis [21]. Both racial discrimination and HIV-related discrimination have predicted low ART adherence among Black/African American individuals living with HIV [22].

Black women’s experience of living with HIV is often compounded by the sexism they face as women and their socialization with traditional female gender roles—socially constructed norms for acceptable female behaviors, attitudes, feelings, thoughts, occupational choices, and personality characteristics [23, 24]. Traditional female gender roles include the coping strategy of self-silencing [25], in which self-needs are not expressed in order to avoid relational loss and conflict. The construct of self-silencing [25] also includes: care-as-self-sacrifice (sacrificing self-needs in order to care for others), divided self (conforming outwardly to gender stereotypes while feeling rebellious internally), and externalized self (judging oneself by external female stereotypic standards, such as media standards of beauty). Among Black women there is an integration of traditional and nontraditional gender roles (e.g. assertiveness) [26, 27] in addition to the Strong Black Woman stereotype, which expects them to “withstand male rejection, economic deprivation, crushing family responsibilities, and countless forms of discrimination” [28]. Researchers found that WLWH (majority Black samples) were more likely to adhere to traditional gender roles compared to women not living with HIV and these roles were associated with higher depressive symptoms, lower ART adherence, and lower viral suppression for WLWH [29–31].

Trauma, discrimination (racism- and HIV- related), and traditional gender roles rooted in sexism share commonalities that may impact ART adherence. Histories of trauma are associated with negative symptoms including avoidance, elevated fear, mistrust, negative views of the self, and not engaging in self-care behaviors [32, 33]. Racial discrimination can range from subtle behaviors to verbal insults or physical assaults and can therefore be traumatic [19, 34]. Racial discrimination is also associated with symptoms of avoidance, guilt/shame, low self-esteem, and fear [34]. Similarly HIV-related discrimination/stigma can include denial of services, insults, and physical assaults and may result in traumatic symptoms [35, 36]. Experiencing trauma, racial discrimination, HIV-related stigma, and being socialized to traditional gender roles (e.g. self-silencing and sacrificing self-needs) [25] may interfere with ART adherence as a self-care behavior [9, 10] among Black women. In addition, due to these adversities women may not view themselves as worthy of longevity/health provided by ART, avoid medical centers or prescribers who may engage in discriminatory behaviors [18], self-silence with prescribers, be distrustful of medications, and have elevated fear around accidental HIV disclosure (and HIV stigma) via pill bottles.

We developed the first intervention for BWLWH to promote medication adherence and ultimately HIV viral suppression. STEP-AD (Striving Towards EmPowerment and Medication Adherence) is an individual intervention that integrates cognitive behavioral therapy (CBT) for trauma symptoms along with strategies for coping with racial and HIV-related discrimination, promoting gender empowerment and positive body image and relationships, and enhancing resilience to decrease trauma symptoms and improve medication adherence. Formative qualitative work [5, 37] with women and community stakeholders informed the development of STEP-AD and indicated that experiences of trauma, racial and HIV-related discrimination, and gender-related stressors were common experiences. Participants expressed a strong desire for STEP-AD, and suggested adaptive coping strategies and content on substance use, body image, and healthy relationships, which were integrated into STEP-AD. Thereafter an open pilot trial (case study series) of STEP-AD among five BWLWH supported the preliminary acceptability and feasibility of the procedures [38]. However, a pilot RCT comparing STEP-AD to a control condition was needed to demonstrate preliminary efficacy and for additional evidence of acceptability/feasibility. We hypothesized that STEP-AD would lead to greater increase in ART adherence and decreased trauma symptoms compared to enhanced treatment as usual (E-TAU) between baseline and 3- (acute) and 6- months (follow-up). In addition, we hypothesized similar results for secondary outcomes of PTSD diagnosis, viral load, and CD4.

## Methods

### Participants

Between October 2017 and July 2019 BWLWH in a metropolitan area in the Southeastern United States were recruited, enrolled, and completed study sessions and follow up visits. All study procedures were approved by the University of Miami Institutional Review Board and the study was registered at ClinicalTrials.gov (NCT02764853). Posters and flyers advertising the study were distributed at community events, community-based organizations, community health centers, clinics, and hospitals. Individuals who were interested in participating contacted our study staff and were screened for eligibility. Women were eligible for an in-person baseline assessment if they met the following inclusion criteria (1) Black and/or African American, (2) ≥ 18 years old, (3) English speaking, (4) Cis-gender woman, (5) History of abuse/trauma, (6) Prescribed ART for at least the last two months, and (7) at risk for suboptimal HIV outcomes as suggested by self-reported detectable viral load within the past year, less than “excellent” ART adherence, and/or missed HIV-related medical visits within the past year.

### Enrollment

The baseline assessment process occurred over two visits (across two weeks). At baseline visit one participants completed the informed consent process, used Research Electronic Data Capture (REDCap, a secure web-based application) [39] to complete self-report surveys, and participated in a semi-structured clinical interview. Participants were given the Wisepill medication-monitoring device and instructed to place their HIV medication inside and take them as they normally would. Two weeks later participants returned for baseline visit two and were given feedback and informed of their eligibility for randomization. In addition to criteria 1 through 6 in the paragraph above, eligibility criteria for randomization were: (a) Low adherence to ART medication (< 80%) or (b) based on medical records detectable viral load within the past year or (c) at-risk for becoming detectable as suggested by missing > / = 1 HIV-related medical visit in the past year. Three exclusion criteria were: (1) Significant untreated mental health issues that would interfere with study participation (e.g., untreated, unstable psychosis or mania) (2) Inability (e.g., due to cognitive or psychiatric difficulties) or unwillingness to provide informed consent and (3) Recent (past 6 months) behavioral treatment for ART adherence or trauma.

### Sample Size

Our aim was not necessarily to find statistically significant group differences with the current RCT. Instead, emphasis in this pilot RCT was to establish acceptability, feasibility, and preliminary efficacy. Therefore, in lieu of a formal a priori power analysis, sample size estimates were based on recommendations by Rounsaville, Carroll, and Onken [40], who suggest that for behavioral pilot RCTs, randomizing between 15 and 30 participants to each condition should be sufficient.

### Randomization

Eligible women participated in the one session Life-Steps [41] intervention prior to being randomized to either STEP-AD or ETAU. Life-Steps is a brief intervention with cognitive-behavioral, problem-solving, and motivational interviewing techniques to promote adherence. The Life-Steps session includes psychoeducation on the benefits of adherence to ART, introduction to strategies to help participants modify non-adaptive cognitions about taking their medications, review of common barriers to adherence, and overview of problem-solving techniques for challenges with adherence.

### Study Visits

*STEP-AD Intervention* After completing the Life-Steps session women randomized to the STEP-AD intervention were asked to attend nine weekly sessions. The content areas covered by each session are as follows: (1) Psychoeducation on the treatment model (2) Substance Use and Enhancing Resilience (3) Trauma Impact (4) Cognitive Restructuring (5) Racial Discrimination Impact and Coping (6) HIV Stigma Impact and Coping (7) Gender-related Stressors and Coping (8) Body Image and Healthy Relationships and (9) Practice, Review, and Relapse Prevention. Additional details on each session are provided in Table 1. During each session women’s adherence in the past week was reviewed (e.g., successes, obstacles, solutions) and the connections between the stressors and adherence were discussed. With a focus on empowerment and strengths, the intervention emphasized that women possess the strength, power, and capacity to improve their lives and health behaviors, cope with adversities, and heal. In between each session women were asked to complete activities consisting of writing, practicing cognitive restructuring, and utilizing adaptive coping strategies (see additional details in [38]). A key cornerstone of the intervention was also self-care, i.e., activities to (a) enhance mood and mental well-being (e.g., spending time with peers) and (b) improve engagement in health care (e.g., scheduling and attending appointments). At each session women planned specific individualized self-care activities to engage in prior to the next session, and the clinician queried about their self-care practice at the following session.

**Table 1:** Overview of STEP-AD Sessions and Content

Sessions	Focus	Between session activities
Universal content across sessions	Medication Adherence—Women utilize an electronic medication adherence box (see methods section) and at the start of each session their adherence is displayed on a graph and discussed (e.g., praises, obstacles, solutions) Self-care—At the end of each session women make a self-care plan that consists of individualized activities they would engage in prior to the next session. Self-care is then review at the start of the following session Empowerment and Strengths—There is an ongoing view and emphasis on the women as possessing the strength, power, and capacity to improve their lives and health behaviors, cope with adversities, and heal	
Session 1: Psychoeducation on the treatment model	Present psychoeducation on trauma/PTSD and the treatment model on the connections between symptoms of trauma/abuse, racial discrimination, and gender related coping with medication nonadherence and how adaptive strategies for addressing trauma, coping with racial discrimination, and enhancing gender empowerment and resilience may moderate effects	1. Essay on substance use and success/challenges Self-care activities 1) fun/mood lifter and 2) engagement in care and adherence
Session 2: Substance Use and Enhancing Resilience	Resilience: Define the resilience concept and its association with medication adherence, discuss coping strategies and techniques (e.g., viewing obstacles as challenges) utilized by resilient individuals following trauma, and assist the participant in eliciting evidence of her resilience and coping strategies to optimize her resilience in terms of adherence Substance Use: Elicit the participant’s past and current substance use, discuss how substance is often used to cope with trauma, discuss strategies participant has used to address substance use, introduce behavioral activation (self-care) as a strategy, and make a plan with participant to address substance use that may occur during this program	1. Essay on impact of trauma 2. Coping with events worksheet Self-care activities
Session 3: Trauma Impact	Conduct an in-depth review of the trauma impact statement, elicit negative cognitions, and introduce cognitive restructuring	1. Rewrite of trauma impact essay 2. Cognitive restructuring sheet 3. Self-care activities
Session 4: Cognitive Restructuring	Continue to teach and practice cognitive restructuring strategies to correct negative thoughts from trauma linked to nonadherence	Essay on the impact of racism Cognitive restructuring sheet. Self-care activities
Session 5: Racial Discrimination Impact and Coping	Review the link between racial discrimination with medication adherence, discuss different strategies for coping with racism, explore coping strategies the participant tends to utilize, assist the participant in weighing the benefits and/or costs of using these various coping strategies, minimize use of nonadaptive strategies, and enhance use of adaptive strategies	1. Essay on impact of HIV stigma/ discrimination 2. Cognitive restructuring sheet 3. Self-care activities 4. Coping with events worksheet
Session 6: HIV Stigma Impact and Coping	Review the link between HIV stigma/discrimination and medication adherence, discuss different strategies for coping with HIV stigma, explore coping strategies the participant tends to utilize, assist the participant in weighing the benefits and/or costs of using these various coping strategies, minimize use of nonadaptive strategies, and enhance use of adaptive strategies	1. Essay on impact of gender-related stressors 2. Self-care activities 3. Cognitive restructuring sheet 4. Coping with events worksheet
Session 7: Gender-related Stressors and Coping	Review gender-related coping strategies that women tend to utilize, minimizing use of strategies that relate to medication nonadherence and enhancing use of adaptive strategies (e.g., self-advocacy)	1. Essay on Body Image 2. Self-care activities 3. Cognitive restructuring sheet 4. Coping with events worksheet
Session 8: Body Image and Healthy Relationships	Body Image: Define body image and discuss how it may be linked with overall feelings of self-worth and self-care and discuss how the participant views her body (from head to toe, including hair) Healthy Relationships: Define health vs. unhealthy relationships and how they may be linked to mood, self-care, etc. and discuss the relationships she has/had (not just intimate), ones she desires, and steps she is taking	Essay on adaptive coping for stressors and enhancing medication adherence 1. Self-care activities 2. Cognitive restructuring sheet 3. Coping with events worksheet
Session 9: Practice, Review, and Relapse Prevention	Continue to reinforce the correction of negative trauma thoughts and adaptive coping strategies for racial and HIV discrimination, gender empowerment, and resilience for better medication adherence Discuss relapse prevention and continue to reinforce adaptive coping and adherence skills	Self-care activities

*ETAU Control* Women randomized to the control condition (following the Life-Steps session) were asked to attend four bi-weekly appointments with a study research associate. Women completed self-report measures and the research associate reviewed their Wisepill medication adherence.

*Baseline and Follow-up Visits* Immediately following the completion of the intervention or ETAU visits women completed an acute follow-up approximately 3 months after baseline (henceforth referred to as T2) and a 3-month follow-up visit at approximately 6 months after baseline (henceforth referred to as T3). All measures completed at baseline were repeated at follow ups.

**Fig. 1:**
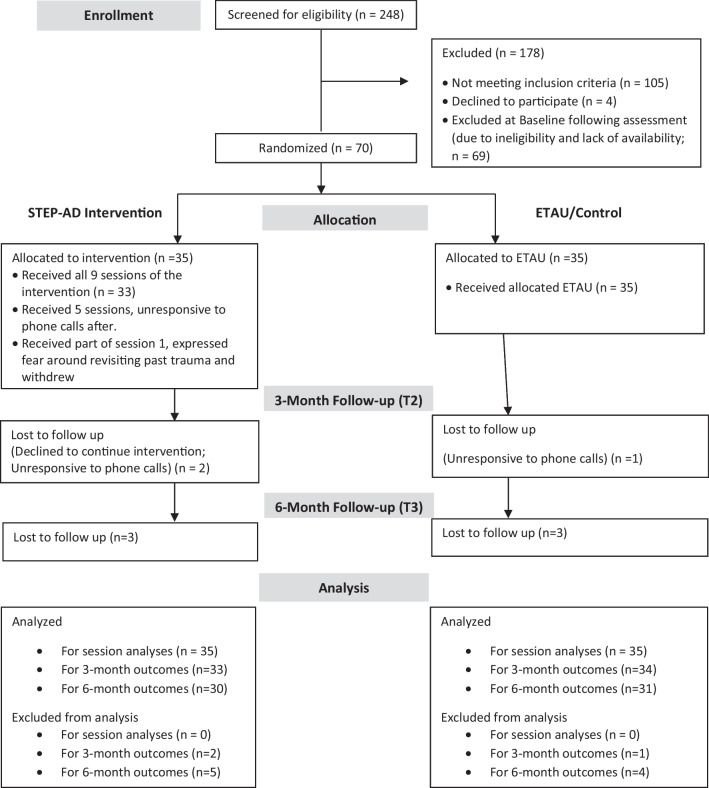
CONSORT Flow Diagram for a Pilot RCT of the STEP-AD intervention among BWLWH and a history of trauma

Women were given $50 total at the baseline assessment ($25 at visit 1 and $25 at visit 2), $25 for each weekly visit in STEP-AD, $50 at each bi-weekly visit in ETAU, and $50 for T2 and T3. A CONSORT flow chart is provided in Fig. 1. Participants primarily interfaced with a team of majority Black women including the Principal Investigator, two research coordinators, one graduate student, and one research coordinator/recruiter who is a BWLWH.

### Independent Assessors

At the follow up visits two independent assessors blind to study condition conducted a clinical diagnostic interview utilizing a structured interview for DSM 5 diagnoses and administered the *Davidson Trauma Scale* [44]. Assessors were a doctoral student in psychology and a master’s level clinician.

### Measures

Outcomes of interest for the current study were sociodemographic variables, electronically monitored ART adherence [45–48], HIV viral load [49], detectability, and suppression, CD4 count [50], DSM-5 PTSD diagnosis, and trauma symptoms [44]. A detailed description of measures used to assess these constructs are found in Table 2.

**Table 2:** Description of Measures Administered throughout the Pilot RCT of STEP-AD

Construct	Measure
Self-report Sociodemographic Survey	Participants shared information including age, annual income, living situation, education level, sexual orientation, country of birth, and years since HIV diagnosis
ART Adherence	Wisepill monitored a single medication which the participant took most frequently, or which they had the most difficulty taking, an approach which demonstrated good feasibility and acceptability among PLWH [44, 45]. At each study visit participants were provided with visual calendars showing what days they took medication from the Wisepill and which days they did not. Consistent with prior studies [46, 47] a day initially noted as ‘medication not taken’ would be changed to ‘taken’ if a participant shared that she definitely took her medication, but not from the Wisepill (e.g. took two pills at the same time [for their one pill a day regimen] from the Wisepill when they planned to spend the night at a friend’s home). For the two weeks preceding the second baseline visit, bi-weekly visits, and the follow-up visits (3 and 6 months), percent ART adherence (days Wisepill was opened ÷ total days) was calculated
HIV viral load, detectability, and suppression	Medical records were reviewed for participant’s viral load within the year preceding baseline. Blood draws were also conducted at baseline and follow ups. When women had completed blood work within 30 days of the visit, those viral load and CD4 (below) results were used. A cutoff of 20 was used for detectable (> / = 20) vs undetectable (< 20) viral load, while a cutoff of 200 was used for viral suppression (< 200) vs viral nonsuppression (> / = 200) [49]
CD4	Assay captured CD4 count from blood collected from participants at baseline and follow ups. CD4 is an indicator of immune function with higher scores indicating better function [48]
Structured interview for DSM-5	A structured interview was administered by a trained clinician (at least master’s degree) to assess for current diagnoses including PTSD based on the Diagnostic and Statistical Manual of Mental Disorders, Fifth Edition (DSM-5)
Davidson Trauma Scale	The DTS [43] is a 17-item measure of post-traumatic stress disorder symptoms (PTSD) that assesses both frequency and severity for each symptom using a 5-point likert scale (e.g., 0 = not at all/ not at all distressing, 4 = everyday/ extremely distressing). Participants are asked to identify the trauma/abuse experience that has been the most disturbing for them and rate their symptoms. A sample item is “Have you ever had painful images, memories, or thoughts of the event?” This scale has demonstrated excellent reliability (Cronbach’s alphas of 0.99) and validity in prior literature [43] and Cronbach’s alpha was 0.85 in the present sample

*Treatment fidelity* A therapist treatment adherence form was created by the principal investigator and used to assess clinician competence and adherence. The form includes 9 session structure ratings that assess techniques (e.g., cognitive restructuring and/or adaptive coping) and content (e.g., review of medication adherence and PTSD symptoms) for each STEP-AD session. Each item is rated using a 7-point Likert scale ranging from 0 (least adherent) to 6 (most adherent). Ratings were made by two clinical psychology PhD students (who were not interventionists) using audio recordings from 20 randomly selected STEP-AD sessions.

*Treatment acceptability* Women who completed the STEP-AD intervention participated in an exit interview at T3 that asked about skills they found helpful or unhelpful, their understanding and thoughts about the content covered, and their thoughts on the length of the intervention and the between session activities.

### Statistical Analyses

SPSS version 26 was used to compute descriptive statistics on sociodemographic characteristics. Qualitative responses from the brief exit interviews given to women in the intervention arm were reviewed and summarized.

STATA 17.0 was used to run difference-in-difference analyses using both linear and nonlinear mixed models in which we allowed the intercept to vary, to capture dependencies among participants within the respective groups. According to Twisk and colleagues [51] when there are differences at baseline between the treatment and control group due to random fluctuations and measurement error, if unaccounted for, then there is a tendency to change the average for the respective groups (treatment and control)—the classical problem of regression to the mean. Thus here, we examine if the intervention study was effective by accounting for baseline differences. The model is given as,

$$H_{it} = \beta_{0} + \gamma_{0} Treated_{i} + \beta_{1} DT_{t} + \gamma_{1} \left( {Treated_{i} \times \, DT_{t} } \right) + \varepsilon_{it} \; i = 1, \ldots ,n{\kern 1pt} {\kern 1pt} and{\kern 1pt} t = 1,2, \ldots ,T.$$

An explanation of the statistical terms in this model is provided in Table 3. For each outcome, three sets of difference-in-difference analyses were conducted: (1) over time from baseline through T3 (2) T2 (3) T3. In addition, for the primary outcome (adherence) and in alignment with literature noting that participants may boost pro-health behaviors (may not be a true reflection of their baseline habits) at the start of a study, a fourth analysis (a) removed the baseline adherence value and began with the next value and (b) examined the difference between condition groups throughout active sessions. Across analyses, the joint significance of (γ_0_ + γ_1_) indicated the overall effectiveness of the treatment.

**Table 3:** Descriptions of statistical terms in difference-in-difference model

Variable	Description
H_it_	Represents the health outcome of individual *i* at time period *t*
Treated_i_	A categorical variable (dummy variable) that takes the value 1 for all the individuals in the treatment group and zero (0) for individuals in the control group. The Treated_i_ variable controls for preexisting mean differences before the intervention between the treated and the control group. Therefore, the coefficient estimate of γ_0_ represents preexisting (baseline) differences between the treated and control group
DT_t_	A categorical variable (dummy/binary variable) that equals to one (1) for the post-baseline period (during and after the intervention) and zero (0) for the pre-treatment period. DT_t_ is used to capture trends, unmeasured variables/activities that affect the health outcomes of all individuals across groups
ε_it_	A random error component that has a mean of zero and constant variance
Treated_i_ × DT_t_	Interaction term that captures additional change during and post intervention, controlling for differences before, during, and after the intervention

## Results

### Participant Characteristics

Clinical and sociodemographic characteristics of the 70 BWLWH who were randomized are presented in Table 4. In brief, 67% of women had completed at least high school/GED, 67% had an annual income of less than $11,999, 63% rented an apartment/home, 41% were in a relationship, and 71% identified as exclusively heterosexual. At baseline 80% had a detectable viral load within the past 12 months (82.9% of STEP-AD and 77.1% of ETAU), 48.57% had a detectable viral load in the past month, 11.83% had ART adherence below 80% in the past two weeks, and 18.57% had missed an HIV-related appointment in the last 12 months. BWLWH in the STEP-AD and ETAU groups did not differ significantly on the vast majority of variables at baseline (see Tables 4 and 5). The flow of women through the study is depicted in Fig. 1. Scores for each study outcome are presented in Table 5 as a function of time and condition.

**Table 4:** Sociodemographic characteristics of BWLWH in STEP-AD versus ETAU

Characteristic		Mean (*SD, range*) or n (%)	
		STEP-AD (n = 35)	ETAU (n = 35)
Age		47.59 (10.90, 22–59)	49.31 (10.68, 22–65)
Education	Eighth grade or Lower	3 (8.6%)	1 (2.9%)
	Some high school	9 (25.7%)	10 (28.6%)
	High school graduate/GED	14 (40%)	14 (40%)
	Some college	7 (20%)	8 (22.9%)
	College graduate	0 (0%)	2 (5.7%)
	Some graduate school	1 (2.9%)	0 (0%)
	Missing	1 (2.9%)	0 (0%)
Income	Less than $5,000	13 (37.1%)	14 (40%)
	$5,000–$11,999	9 (25.7%)	11 (31.4%)
	$12,000–$15,999	3 (8.6%)	7 (6.9%)
	$16,000–$24,999	1 (2.9%)	3 (8.6%)
	$25,000–$34,999	0 (0%)	1 (2.9%)
	$35,000–$49,999	0 (0%)	0 (0%)
	$50,000 and greater	1 (2.9%)	0 (0%)
	Choose not to answer or Don’t know	7 (20%)	6 (17.2%)
	Missing	1 (2.9%)	0 (0%)
Employment status	Full-time Work	1 (2.9%)	1 (2.9%)
	Part-time Work	1 (2.9%)	4 (11.4%)
	Full or Part-time School	1 (2.9%)	3 (8.6%)
	Neither Working or in School	9 (29.7%)	7 (20%)
	On Disability	19 (54.3%)	21 (60%)
	Other	2 (5.7%)	2 (5.7%)
	Missing	3 (8.6%)	1 (2.9%)
Housing arrangement	Renting home or apartment	21 (60%)	23 (65.7%)
	Owned by you or someone else in household	4 (11.4%)	5 (14.3%)
	Publicly subsidized housing	4 (11.4%)	3 (8.6%)
	A friend or relative’s home/apartment	2 (5.7%)	2 (5.7%)
	Homeless: sleeping in a shelter	2 (5.7%)	0 (0%)
	Homeless: sleeping on the street, beach, car	0 (0%)	1 (2.9%)
	Missing	1 (2.9%)	1 (2.9%)
Living situation	Lives with Self	20 (57.1%)	16 (45.7%)
	Partner/spouse	5 (14.3%)	8 (22.9%)
	Roommates	3 (8.6%)	0 (0%)
	Children	8 (22.9%)	11 (31.4%)
	Group Home or Residential Treatment	0 (0%)	0 (0%)
	Other	5 (14.3%)	8 (22.9%)
	Missing	1 (2.9%)	1 (2.9%)
Place of birth	U.S. Born	34 (97.1%)	32 (91.4%)
	Non-U.S. Born	1 (2.9%)	3 (8.6%)
Parents of children		26 (74.3%)	31 (88.6%)
	Missing	1 (2.9%)	1 (2.9%)
Number of children of parents of children		2.96 (1.97, 1–9)	2.77 (1.50, 1–6)
	Missing	0 (0%)	0 (0%)
Religion	Christian	11 (31.4%)	5 (14.3%)
	Catholic	2 (5.7%)	1 (2.9%)
	Baptist	17 (48.6%)	23 (65.7%)
	None	2 (5.7%)	3 (8.6%)
	Other	1 (2.9%)	2 (5.7%)
	Missing	1 (2.9%)	0 (0%)
Relationship status	Married	3 (8.6%)	5 (14.3%)
	Cohabiting relationship, unmarried	4 (11.4%)	7 (20%)
	Non-cohabiting relationship	7 (20.0%)	3 (8.6%)
	Single	11 (31.4%)	17 (48.6%)
	Divorced/Separated	5 (14.3%)	3 (8.6%)
	Widow or Loss of Partner	2 (5.7%)	0 (0%)
	Missing	3 (8.6%)	0 (0%)
Sexual orientation*	Exclusively heterosexual	21 (60%)	29 (82.9%)
	Heterosexual, some same gender loving experience	3 (8.6%)	3 (8.6%)
	Bisexual	2 (5.7%)	2 (5.7%)
	Exclusively gay/same gender loving	4 (11.4%)	0 (97.1%)
	Missing	5 (14.3%)	1 (2.9%)

*Indicates a significant difference in sexual orientation (p < 0.05) between the STEP-AD and ETAU groups at baseline. There were four exclusively lesbian/gay women in the STEP-AD condition compared to zero in ETAU. However, the number of bisexual and heterosexual women with same gender loving experience women were equivalent in both conditions

**Table 5:** Outcome values across T1, T2, and T3 for STEP-AD versus ETAU

Outcome	Mean (*SD, range*) or n (%)		
	Baseline/T1	T2 (3 month follow up)	T3 (6 month follow up)
Mental health			
Post-traumatic stress disorder diagnosis—current			
STEP-AD	19 (54.29%)	4 (12.12%)	3 (9.09%)
ETAU	17 (48.57%)	10 (29.41%)	9 (28.12%)
PTSD symptoms (DPS/clinician administered)			
STEP-AD	42.68 (26.48)	20.26 (24.82)	18.70 (20.51)
ETAU	42.97 (31.03)	30.55 (33.63)	32.06 (31.54)
HIV health and related behaviors			
ART adherence—past 2 weeks Wisepill			
STEP-AD	95.51 (10.11)	95.88 (11.08)	95.24 (17.67)
ETAU	87.74 (27.55)	90.18 (25.87)	88.81 (24.53)
HIV viral load linear			
STEP-AD	3315.37 (8970.35)	4767.30 (15,701.4)	3118.69 (11,156.42)
ETAU	7222.89 (22,359)	9258.34 (26,757.57)	8178.16 (32,038.15)
HIV viral load log			
STEP-AD	2.01 (1.12)	1.92 (1.12)	1.88 (1.01)
ETAU	2.16 (1.19)	2.21 (1.30)	1.95 (1.19)
VL detectable			
STEP-AD	14 (40.0%)	13 (40.62%)	14 (42.42%)
ETAU	20 (57.14%)	17 (50%)	14 (43.75%)
VL suppression			
STEP-AD	26 (74.29%)	24 (75%)	24 (75%)
ETAU	26 (74.29%)	23 (67.65%)	26 (78.79%)
CD4 absolute count			
STEP-AD	717.85 (363.23)	696.15 (374.29)	725.94 (354.74)
ETAU	553.15 (392.23)	528.06 (347.78)	528.53 (357.14)

*Indicates a significant difference between the STEP-AD and ETAU groups at baseline

### Retention

Of the 70 women randomized, 67 (96%) were retained through T2 and 65 (93%) were retained through T3. Thirty-three (94%) of the women randomized to STEP-AD completed all nine intervention sessions.

### Therapist Adherence

In general, the clinician was highly adherent to the treatment manual, with an average percent score of 95% (SD = 6) for the random sample of 20 intervention sessions rated.

### Evidence of Acceptability from Exit Interviews

Twenty-nine of the women who completed the STEP-AD intervention participated in the exit interview at T3. Overall women shared very positive reviews (see quotes in Table 6) of the intervention. For instance, women shared their satisfaction with the skills and coping strategies taught, the content covered, the focus on strengths/empowerment of BWLWH, and the clinician’s approach. Women had varying feelings about the between session activities initially, but overall valued it. In addition, several women expressed a desire for more sessions.

**Table 6:** Exit interview quotes from BWLWH who completed the STEP-AD intervention arm

Skills and coping	
Cognitive restructuring	“What you could do when you get negative thought…I could have a negative thought but I don’t hold it too long”
	“Thinking about the pros and cons and the positive and negatives.”
Emotional regulation/ mindfulness	“I especially [liked] the part where you take time to you know breathe and think about your responses to some things.”
Self-care practices	“The self-care [skill] was the best one”… “taking care of yourself, taking time for yourself”
Intervention content	
Overall content	“All of them [sessions] was important so there was none that’s most important, all of them was important and educational”
	“It motivated me to do better, motivated me to want to keep doing better, all in all it just helped me with things that I was going through in my life.”
	“I want to hear about what empowers a Black woman, and what inspires a woman.”
Processing trauma	“Letting go of the trauma and talking about it and coping with it and trying to cope with how to live with, how to live with dealing with the drugs and self-discipline. I slowed down a lot, I slowed down a tremendous (inaudible), with substance abuse.”
Body image	“By her doing that it allowed me to look in the mirror and say you are a beautiful Black woman.”
Health relationships	“You’ve got to love you first in order for others to love you [and] you have to accept me for who I am and if you can’t there’s no need for us being together because I love me for who I am.”
Discrimination & stigma	“It’s good that you can talk about stigma” and “coping with HIV and coping with the stigma [against] being Black”
Medication adherence	“It makes you better aware of actually taking your medications and being persistent with it”
	“It taught me about how to stay in control of my medicine, not miss it.”
Between session activities	“It was hard to get into it but once I start writing it just flowed out. Especially when she made me write the essays, you know it made me go deep and really express or think about what I was holding onto. So those were really good for me.”
	“The homework was really amazing…to go back and look over some of my work…I constantly still do. It gave me an opportunity to not depend on the clinician, I gotta depend on myself for my work, I gotta work on me on a daily basis.”
Clinician’s approach	“She did excellent, I wish she had more sessions”
	“She’s a really good listener.”
	“I’d be boo-hoo crying and snotting and stuff, but she will gently lean forward and just push all the work aside and just talk woman to woman and make me realize that you know you don’t have to, what you are going through she will break it down to where I can understand why and then making me look at the goodness that I have within me she would bring it out and bring it out, and before you know it we’re laughing again.”
Research program staff and environment	“It helped me look at myself differently as a Black woman because you don’t really go places where it’s multiple Black people.”
	“You guys listened, and I could just tell like it’s not about the money it’s about y’all wanting to help people.”

*Skills and coping* Women discussed their satisfaction with skills including cognitive restructuring and thinking of the ABCs (antecedents, behaviors, and consequences), emotion regulation, scheduling and prioritizing self-care.

*Intervention content* Women repeatedly shared how they were satisfied with all the content areas covered (e.g., resilience/empowerment, trauma processing, body image, healthy relationships, HIV stigma, etc.) and how it had positively impacted them. A participant explained how the treatment model presented in the first session had an impact: “This treatment model put it all in perspective. The impact [of adversities], the invalidated self, resulting in lack of self-care. But then her teaching me what to do was a good thing.” Similarly, women shared how content on positive body image and healthy relationships impacted them. One woman explained the effect of being asked to reflect on and challenge cognitions about her body and another woman linked the content on healthy relationships to enhanced self-love and expectations in relationships. Further, women were satisfied with the content on coping with discrimination and stigma. Lastly, women appreciated revisiting medication adherence at each session and its connection to the various adversities. Women had mixed reviews about being asked to do activities between sessions, but ultimately valued the skills learned.

*Clinician’s approach* Across the board women shared how satisfied they were with the clinician’s client-centered approach that made them feel heard.

*Research program staff and environment*. Women also expressed satisfaction about the study team’s gender and racial composition reflecting the women’s identities as Black women. In addition, women felt that there was emphasis placed on valuing and serving them.

*ART medication adherence* Women in STEP-AD had significantly higher adherence than women in ETAU across the active treatment sessions (z = 2.01, p = 0.045) and showed a trend of higher adherence over time (baseline through T3) (z = 1.90, p = 0.06) (see Tables 7 and 8). However, women in STEP-AD did not have significantly higher adherence compared with women in ETAU specifically at T2 (z = 1.16, p = 0.25) and T3 (z = 1.19, p = 0.23) when active sessions adherence data was excluded.

**Table 7:** Difference-in-difference findings on continuous outcomes

Outcome	Coefficient	Standard error	z	P > IzI	95% confidence interval
Wisepill Adherence (past 2 weeks) through session 9	9.36	4.66	2.01	0.045*	0.23 18.48
Wisepill Adherence (past 2 weeks) overtime	8.09	4.25	1.90	0.06^t^	− 0.25 16.43
T2	5.65	4.86	1.16	0.25	− 3.87 15.17
T3	6.09	5.10	1.19	0.23	− 3.91 16.08
VL overtime	− 4475.46	4643.62	− 0.96	0.34	− 13,576.79 4625.87
T2	− 4397.84	4680.16	− 0.94	0.35	− 13,570.79 4775.10
T3	− 4575.52	4897.63	− 0.93	0.35	− 14,174.71 5023.66
VL log overtime	− 0.11	0.24	− 0.44	0.66	− 0.59 0.37
T2	− 0.19	0.28	− 0.67	0.51	− 0.75 0.37
T3	− 0.007	0.27	− 0.02	0.98	− 0.54 0.53
CD4 overtime	161.26	83.32	1.94	0.05^#^	− 2.04 324.56
T2	152.44	86.62	1.76	0.08	− 17.33 322.22
T3	170.11	86.76	1.96	0.05^#^	0.06 340.16
PTSD Symptoms overtime	− 10.96	6.18	− 1.77	0.08^t^	− 23.08 1.16
T2	− 10.24	7.07	− 1.45	0.15	− 24.10 3.61
T3	− 12.43	6.73	− 1.85	0.07^t^	− 25.62 0.76

*p < 0.05. ^t^p < 0.10. For CD4 findings ^#^p = 0.05

**Table 8:** Difference-in-difference findings on dichotomized outcomes

Outcome	Odds ratio	Standard error	z	P > IzI	95% confidence interval
PTSD diagnosis overtime	0.07	0.08	− 2.19	0.03*	0.01 0.76
T2	0.07	0.09	− 1.96	0.05*	0.005 0.99
T3	0.06	0.09	− 1.86	0.06^t^	0.003 1.15
VL detectable overtime	0.83	0.52	− 0.29	0.77	0.24 2.87
T2	0.61	0.58	− 0.52	0.61	0.09 3.95
T3	1.02	0.73	0.03	0.98	0.25 4.13
VL suppression overtime	1.25	1.03	0.27	0.79	0.25 6.34
T2^&^					
T3	1.09	1.01	0.09	0.93	0.18 6.64

*p < 0.05. ^t^p < 0.10. ^&^Did not converge

*HIV viral load, detectability, and suppression* When descriptively examining viral suppression among women (n = 9 in STEP-AD, n = 9 in ETAU) who had non-suppression at baseline, 57% (n = 6) of women in STEP-AD and 33% (n = 3) of women in ETAU had viral suppression at T2. However, via difference-in-difference analyses women in the STEP-AD condition did not significantly differ from women in the ETAU condition over time or specifically for T2 or T3 on HIV viral load (Over time: z = − 0.96, p = 0.34; T2: z = − 0.94, p = 0.35, T3: − 0.93, p = 0.35), detectable viral load (Over time: OR = 0.83, z = − 0.29, p = 0.77; T2: OR = 0.61, z = − 0.52, p = 0.61; T3: OR = 1.02, z = 0.03, p = 0.98), or viral load suppression (Over time: OR = 1.25, z = 0.27, p = 0.79; T2: no convergence; T3: OR = 1.09, z = 0.09, p = 0.93).

*CD4* When controlling for baseline differences, women who completed STEP-AD compared to E-TAU had borderline significantly higher CD4 count over time (z = 1.94, p = 0.05). When T2 and T3 were examined separately women in STEP-AD had borderline significantly higher CD4 count at T3 (z = 1.96, p = 0.05) but not T2 (z = 1.76, p = 0.08).

*PTSD clinician diagnosis* Women in the STEP-AD intervention compared to women in the ETAU condition had significantly lower likelihood of a PTSD diagnosis over time (OR = 0.07, estimate = − 2.66, p = 0.03). When analyses were conducted for T2 and T3 separately, findings showed that women in STEP-AD had significantly lower PTSD likelihood at T2 (OR = 0.07, z = − 1.96, p = 0.05) and a trend of lower likelihood of PTSD at T3 (OR = 0.06, z = − 1.86, p = 0.06). Descriptively, 54.29% of STEP-AD women and 48.57% of ETAU women began with a PTSD diagnosis at baseline, however fewer women in STEP-AD had PTSD at T2 (12.12% of STEP-AD vs 29.41% of ETAU) and T3 (9.09% of STEP-AD vs 28.12% of ETAU).

*PTSD symptoms* There was a trend of lower PTSD symptoms (measured by Davidson Trauma Scale) over time (z = − 1.77, p = 0.08) for women in STEP-AD compared to ETAU. In addition, there was a trend of lower PTSD symptoms among STEP-AD women at T3 (z = − 1.85, p = 0.07), but no trend or significant finding at T2 (z = − 1.45, p = 0.15). These trends are consistent with descriptive average symptoms among women at T2 (STEP-AD = 20.26, ETAU = 30.55) and T3 (STEP-AD = 18.70, ETAU = 32.06).

## Discussion

The current study conducted a pilot RCT to assess preliminary efficacy and access acceptability and feasibility for STEP-AD, a novel intervention to improve medication adherence among BWLWH. There is currently no other intervention that has been developed to improve medication adherence among BWLWH that integrates both evidence-based strategies and coping strategies informed by BWLWH and community stakeholders to enhance coping around intersectional adversities faced by BWLWH (trauma, racism, HIV-discrimination, and gender-related stressors). STEP-AD is thereby both innovative and necessary given the disproportionate impact of HIV on Black women and disparities along the HIV treatment cascade when BWLWH are compared to women of other racial/ethnic groups.

We present promising findings, in our sample consisting of only 70 BWLWH, that STEP-AD significantly increased ART adherence during the active intervention period, decreased the likelihood of a PTSD diagnosis, and improved CD4 count over time (baseline through T3). In addition, we saw trends (p < 0.10) of STEP-AD improving ART adherence over time and decreasing PTSD symptoms (assessed via Davidson Trauma Scale) over time and at T3. The impact on adherence may have been most felt during weekly sessions when the client and therapist were engaging in conversations about stressors, coping, skills, and adherence. Nonetheless, the trend of improvement in ART adherence from baseline through the last follow-up suggests that STEP-AD may have continued to have an effect, however a study with a larger sample size is needed. Interestingly we observed a significant decrease in PTSD diagnoses (assessed via a structured clinical interview for diagnosing mental health), but a trend with PTSD symptoms assessed with a scale indicating the benefits of assessing clinical diagnoses. Also, while the average CD4 count at baseline was higher among women in STEP-AD, the difference-in-difference methodology controlling for baseline differences still indicated that over time women in STEP-AD had higher improvements in CD4 count compared to women in ETAU. This suggests that STEP-AD may improve immune function through the observed improvements in adherence and potentially via biological pathways between mental health improvement (i.e., PTSD) and immune function. Lastly, with caution, it is noteworthy that secondary analyses with viral load over time and at both follow-ups showed anticipated negative values (lower among STEP-AD group) with p-values of 0.34, 0.35 and 0.35. Descriptively, of the 18 women who began the study with viral non-suppression 57% of those in STEP-AD were virally suppressed at follow-up compared to 33% women in ETAU. Perhaps with a large sample that is adequately powered the true efficacy of STEP-AD on viral suppression may be assessed.

Beyond the quantitative outcomes, exit interviews with women who completed STEP-AD indicated that women were highly satisfied with the skills and coping strategies taught, content covered, strengths-based focus, and the clinician’s client-centered approach. Further, women saw the value in between sessions activities and some women wished for additional sessions. This provides additional support for STEP-AD’s acceptability and feasibility among BWLWH.

Our findings from this STEP-AD pilot RCT extend preexisting literature of interventions among WLWH and histories of trauma [52–55] that (a) excluded content on intersectional adversities that are a daily reality for BWLWH [8] and have been linked to ART adherence [9, 10] and (b) did not address adherence or demonstrate improvements. In addition, the Life-Steps single session [55], which both the intervention and control conditions received prior to randomization, has previously shown modest effects on adherence, however women in STEP-AD had higher increases in ART adherence suggesting that STEP-AD provides key content to improve adherence among BWLWH. A large-scale randomized control trial is needed to assess the efficacy of STEP-AD in improving ART adherence and ultimately viral suppression.

**Fig. 2:**
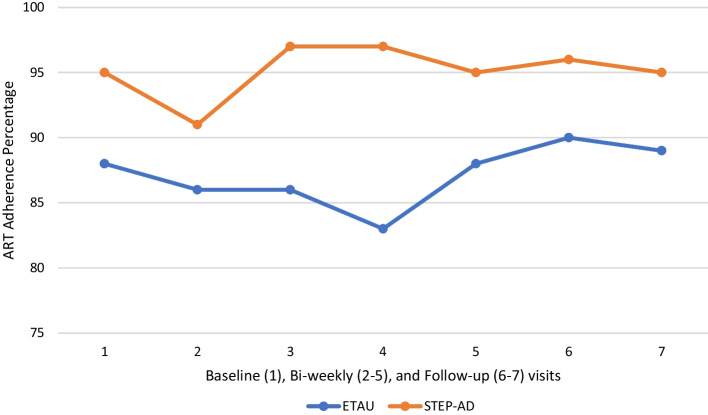
ART Adherence for STEP-AD and ETAU Conditions at Biweekly Visits and Follow-ups

Despite our findings, a few limitations should be noted. First, our sample size of 70 (35 per arm) likely limited our power to detect additional significant findings. Second, STEP-AD was developed specifically for BWLWH and a history trauma and this pilot RCT was conducted in Miami, FL which may limit generalizability. However, the prior open pilot of STEP-AD was conducted in Boston, MA and showed preliminary acceptability and feasibility [38] (Fig. 2).

In conclusion, our pilot RCT of STEP-AD among BWLWH and a history of trauma demonstrated evidence of acceptability, feasibility, and preliminary efficacy with women in STEP-AD compared to ETAU having greater increase in ART adherence, decrease in PTSD diagnosis, and increase in immune function. Additionally, exit interviews indicated that women liked the content, skills, and focus on empowerment. BWLWH continue to experience HIV-related health inequities due to intersectional oppression and unique stressors and STEP-AD is a novel intervention with the potential to improve medication adherence, mental health, and immune function.
